# A systematic review of lenvatinib and sorafenib for treating progressive, locally advanced or metastatic, differentiated thyroid cancer after treatment with radioactive iodine

**DOI:** 10.1186/s12885-019-6369-7

**Published:** 2019-12-12

**Authors:** Nigel Fleeman, Rachel Houten, Marty Chaplin, Sophie Beale, Angela Boland, Yenal Dundar, Janette Greenhalgh, Rui Duarte, Aditya Shenoy

**Affiliations:** 1 Liverpool Reviews & Implementation Group (LRiG), Department of Health Services Research, Institute of Population Health Sciences, University of Liverpool, Whelan Building, Liverpool, L69 3GB UK; 2 The Clatterbridge Cancer Centre NHS Foundation Trust, Bebington, Wirral UK

**Keywords:** Thyroid cancer, Systematic review, Clinical effectiveness, Lenvatinib, Sorafenib, Tyrosine kinase inhibitor

## Abstract

**Background:**

Treatment with radioactive iodine is effective for many patients with progressive, locally advanced or metastatic, differentiated thyroid cancer. However, some patients become refractory to treatment. These types of patients are considered to have radioactive iodine refractory differentiated thyroid cancer (RR-DTC).

**Methods:**

We searched Embase, MEDLINE, PubMed and the Cochrane Library from January 1999 through January 2017. Reference lists of included studies and ongoing trial registries were also searched. Reports of randomized controlled trials (RCTs), prospective observational studies, and systematic reviews/indirect comparisons were eligible for inclusion. In the absence of direct clinical trial evidence comparing lenvatinib versus sorafenib, we assessed the feasibility of conducting an indirect comparison to obtain estimates of the relative efficacy and safety of these two treatments.

**Results:**

Of 2364 citations, in total, 93 papers reporting on 2 RCTs (primary evidence), 9 observational studies and 13 evidence reviews (supporting evidence) were identified. Compared to placebo, RCT evidence demonstrated improvements with lenvatinib or sorafenib in median progression-free survival (PFS) and objective tumour response rate (ORR). Overall survival (OS) was confounded by high treatment crossover (≥75%) in both trials. Adverse events (AEs) were more common with lenvatinib or sorafenib than with placebo but the most common AEs associated with each drug differed. Primarily due to differences in the survival risk profiles of patients in the placebo arms of the RCTs, we considered it inappropriate to indirectly compare the effectiveness of lenvatinib versus sorafenib. ORR and AE findings for lenvatinib and sorafenib from the supporting evidence were broadly in line with RCT evidence. Health-related quality of life (HRQoL) data were limited.

**Conclusions:**

Lenvatinib and sorafenib are more efficacious than placebo (a proxy for best supportive care) for treating RR-DTC. Uncertainty surrounds the extent of the impact on OS and HRQoL. Lenvatinib could not reliably be compared with sorafenib. Choice of treatment is therefore likely to depend on an individual patient’s circumstances.

## Background

Thyroid cancer accounts for approximately 1% of all new malignancies in the United Kingdom (UK) [1] and approximately 3% of all new malignancies in the United States (US) [2]. Commonly asymptomatic and so often discovered incidentally [3], the most common type of thyroid cancer is differentiated thyroid cancer (DTC). A review of 2936 US patients registered with DTC found papillary carcinoma (PTC), follicular carcinoma (FTC) and Hürthle cell carcinoma to constitute 86, 10 and 4% of cases respectively [4]. Globally, DTC incidence is increasing [5]. In part, this increase has been attributed to improved diagnostic and detection techniques [6].

Surgery followed by daily oral medication (levothyroxine**)** to suppress blood thyroid stimulating hormone (TSH) levels is the mainstay of treatment for DTC [7–10]. Additional treatment in the form of radioactive iodine may be required for patients who develop local, regional or metastatic disease (5 to 20% patients [7, 9]). For most patients, radioactive iodine treatment is effective. However, 5 to 15% [4, 11–15] of people with DTC develop radioactive iodine refractory differentiated thyroid cancer (RR-DTC), i.e. they are unable to safely tolerate treatment or they develop DTC that has become resistant to treatment.

For patients with RR-DTC, treatment options have been limited. Chemotherapy is rarely or never recommended by the authors of clinical guidelines [7–10] and thus, for many patients, best supportive care (BSC) has been the only treatment option. However, the authors of published clinical guidelines have noted the promise of targeted therapies including tyrosine kinase inhibitors (TKIs). Lenvatinib is the most recent TKI to be licensed for treating RR-DTC, receiving a licence in the US in February 2015 [16] and in the European Union (EU) in May 2015 [17]. The only other licensed TKI is sorafenib, which was licensed for the treatment of RR-DTC in the US in November 2013 [18] and in the EU in January 2015 [19]. The authors of the US National Comprehensive Cancer Network (NCCN) guidelines now recommend that lenvatinib and sorafenib should be considered for treating progressive and/or symptomatic RR-DTC [10]. The authors, however, caution against their use for patients with stable or slowly progressive indolent disease [10]. The authors of the American Thyroid Association (ATA) guidelines caution that patients who are candidates for TKI therapy “should be thoroughly counseled on the potential risks and benefits of this therapy as well as alternative therapeutic approaches including best supportive care” [7]. Important risks associated with lenvatinib highlighted by regulatory agencies [16, 17] include: hypertension; cardiac dysfunction; arterial thromboembolic events; hepatotoxicity, renal failure or impairment; proteinuria; diarrhea; fistula formation and gastrointestinal perforation; QT interval prolongation; hypocalcemia; reversible posterior leukoencephalopathy syndrome; hemorrhagic events; impairment of TSH suppression/thyroid dysfunction; wound healing complications; and embryo-fetal toxicity. Important risks associated with sorafenib highlighted by regulatory agencies [18, 19] include: dermatologic toxicities including severe skin adverse events (AEs) and hand-foot syndrome; hypertension; posterior reversible encephalopathy syndrome; hemorrhage (including lung hemorrhage, gastrointestinal hemorrhage and cerebral hemorrhage); arterial thrombosis (myocardial infarction); congestive heart failure; QT interval prolongation; squamous cell cancer of the skin; gastrointestinal perforation; symptomatic pancreatitis and increases in lipase and amylase; hypophosphatemia; renal dysfunction; interstitial lung disease-like events; drug-induced hepatitis; impairment of TSH suppression; and embryo-fetal toxicity.

While lenvatinib and sorafenib are available for treating RR-DTC in several countries, the extent to which they are available to patients has varied. For example, lenvatinib and sorafenib are available for all patients who require these treatments in Scotland via the National Health Service (NHS) [20, 21]. However, prior to August 2018, they were only available for patients in special circumstances in the NHS in England. In order to be routinely used in the NHS in England, a positive recommendation from the National Institute for Health and Care Excellence (NICE) is required. We, the Liverpool Reviews and Implementation Group (LRiG), were commissioned, in our capacity as an independent Assessment Group, to provide an independent review of the clinical and cost effectiveness evidence as part of a NICE multiple technology appraisal (MTA). In this paper, we report our systematic review of the clinical effectiveness evidence for lenvatinib and sorafenib and discuss how the evidence has impacted on NICE recommendations for clinical practice.

## Methods

Our systematic review protocol was registered with PROSPERO, the international prospective register of systematic reviews (registration number CRD42017055516). The review was conducted in accordance with the Centre for Reviews and Dissemination (CRD) published guidance on conducting systematic reviews in healthcare [22] and the review is reported in accordance with the Preferred Reporting Items for Systematic Reviews and Meta-Analyses (PRISMA) guidelines [23].

### Search methods for identification of studies

On 10 January 2017, four electronic databases (Embase (Ovid), MEDLINE (Ovid), PubMed and the Cochrane Library) were searched for studies published since 1 January 1999. On 16 May 2017, the clinicaltrials.gov website (a service of the US National Institutes of Health), the International Clinical Trials Registry Platform and the European Union Clinical Trials Register, were searched for information on studies in progress. To identify relevant studies, a combination of index terms for the disease (e.g. thyroid neoplasms) and free text words (e.g. lenvatinib or Lenvima or E7080 or Sorafenib or Nexavar or bay439006) were employed. The database searches were limited to human research and English language studies. No other search restrictions were applied. The search strategies employed are provided in Additional file 1: Online Resource 1.

Evidence submissions from the sponsors of lenvatinib [24] and sorafenib [25] that were submitted to NICE as part of the MTA process were considered for inclusion in our review. The lists of references from the company submissions and all relevant studies identified via the literature searches were cross-checked to identify any papers not identified by the electronic searches.

### Study selection and data extraction

Randomized controlled trials (RCTs), prospective observational studies and systematic reviews/indirect comparisons (hereafter referred to as evidence reviews) of lenvatinib or sorafenib were selected for inclusion in the review. To be included, the population must have included adults with progressive, locally advanced or metastatic thyroid cancer refractory to radioactive iodine, of which at least a subgroup of patients had RR-DTC. A summary of the a priori inclusion and exclusion criteria are provided in Table 1.

**Table 1:** Inclusion / exclusion criteria

Criteria	Inclusion	Exclusion
Patient population	Adults with progressive, locally advanced or metastatic, differentiated thyroid carcinoma, refractory to radioactive iodine	Patients with other types of thyroid cancer or diseases
Interventions	Lenvatinib or sorafenib monotherapy (or in combination with best supportive care)	Lenvatinib or sorafenib in combination with other agents
Comparators^a^	Lenvatinib or sorafenib monotherapy (or in combination with best supportive care), best supportive care, placebo	A comparator other than lenvatinib, sorafenib, best supportive care, placebo
Outcomes	The outcome measures to be considered include: overall survival, progression-free survival, response rate, adverse effects of treatment, health-related quality of life	No study was excluded based on outcomes
Study design	Randomized controlled trials, systematic reviews,^b^ prospective observational studies	Retrospective cohort studies, case series, case reports, comments, letters, editorials, in vitro, animal, genetic or histochemical studies

^a^Where studies included a comparator arm

^b^At the inclusion stage, published reports of indirect comparisons were also included if the indirect comparison was based on RCT evidence, even if the conduct of a systematic review was not reported alongside the indirect comparison

Two reviewers independently screened all titles and abstracts (screening stage 1). Full-text articles of all potentially relevant citations identified during screening stage 1 were retrieved and assessed for eligibility based on the inclusion criteria (screening stage 2). Where necessary, any discrepancies or uncertainties were resolved by discussion or consultation with a third reviewer.

Two reviewers independently extracted and checked data by using a pre-tested data extraction form. Data were extracted relating to study design, patient characteristics and outcomes for RCTs and observational studies and the number and type of studies included, type of analysis conducted and the overall findings/conclusions for evidence reviews. For all study types, data reported in multiple publications were extracted and reported as a single study.

### Quality assessment

The quality of included RCTs and evidence reviews was assessed according to the criteria set out in the Centre for Review and Dissemination’s Guidance [22] for undertaking reviews in healthcare. Two reviewers independently assessed the quality of these studies and, where necessary, disagreements were resolved by consultation with a third reviewer. In accordance with the protocol, quality assessment of the prospective observational studies was not conducted.

### Data synthesis

Data from the included RCTs were considered to provide primary clinical effectiveness evidence. Data from observational studies and from evidence reviews were considered to provide supporting evidence.

## Results

### Literature search and screening

**Fig. 1:**
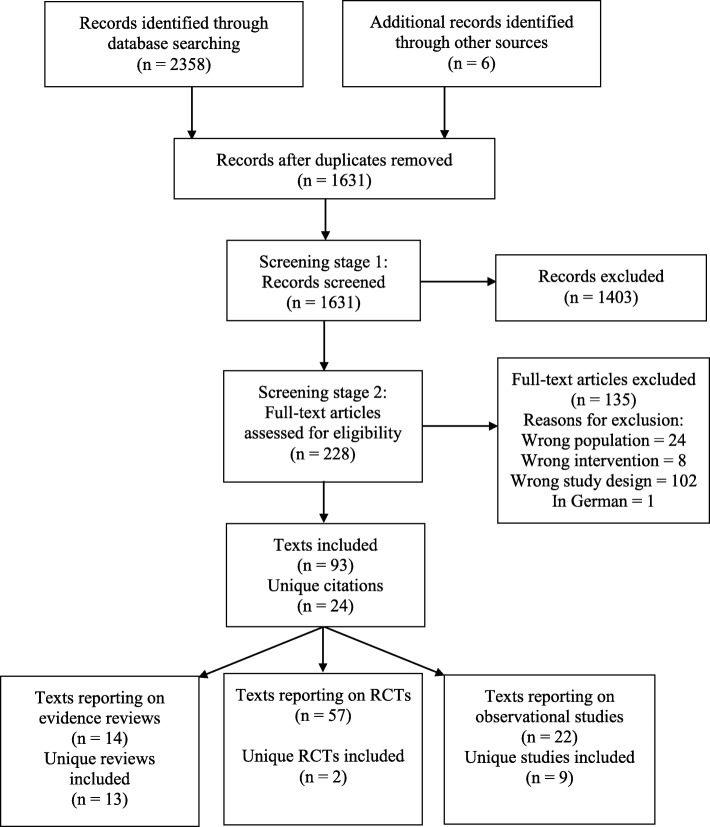
PRISMA flow diagram: studies included in systematic review

The process of study selection is shown in Fig. 1. The electronic database searches yielded 2358 papers and six additional references were identified through searches of the other sources. In total, 93 papers reporting on 24 separate studies and reviews were identified. These included two RCTs [the SELECT trial [26] and DECISION trial [27]], nine prospective observational studies [28–36] and 13 evidence reviews [24, 25, 37–47].

For the RCTs, in addition to the primary published papers [26, 27], data were extracted from other sources identified from the searches, as appropriate. In this paper, additional information for the SELECT trial was extracted from the company submission from Eisai Ltd. [24], the clinical study report (CSR) (unpublished), three conference abstracts [48–50] and the European public assessment report (EPAR) for lenvatinib [51]. For the DECISION trial, additional information was extracted from the company submission from Bayer HealthCare [25], an additional published paper with supplementary safety data [52], the CSR (unpublished), three conference abstracts [53–55] and the EPAR for sorafenib [56].

For one of the included prospective observational studies of sorafenib, known as UPCC-03305 [32], the majority of data were extracted from later conference reports of the same study [57–59] which reported baseline characteristics from a greater number of patients [58], efficacy data [59] and safety data [57].

### Characteristics of included studies

#### Characteristics of randomized controlled trials (primary evidence)

Both of the included RCTs [26, 27] were phase III multi-centre double-blind trials designed to compare the intervention of interest (lenvatinib or sorafenib) with placebo. Subjects were randomized 2:1 to the intervention and comparator arms of the SELECT trial (lenvatinib, *n* = 261; placebo, *n* = 131) [26] and 1:1 in the DECISION trial (sorafenib, *n* = 207; placebo, *n* = 210) [27]. Both trials permitted some concomitant therapies (such as TSH suppression) in both the intervention and placebo arms. Thus, the placebo arm in both trials could be considered to be equivalent to BSC. The types of concomitant therapies were broadly similar in both trials. However, a potentially important difference between the two trials was that palliative radiotherapy, which is commonly available as part of BSC in clinical practice, was only permitted in the DECISION trial, not the SELECT trial. Nonetheless, rates of palliative radiotherapy administered to patients in the DECISION trial were relatively low: 10.6% of patients treated with sorafenib and 21.4% of patients treated with placebo [25].

Patients were eligible to receive treatment (intervention or placebo) in both the SELECT and DECISION trials until disease progression [26, 27]. In both trials, patients were then enrolled into open extension phases [24, 25]. In the DECISION trial, patients who had progressed on sorafenib were permitted to continue to receive sorafenib until further disease progression and approximately a quarter (26.6%) of patients did so [53, 54]. In both the SELECT and DECISION trials, patients in the placebo arms could cross over from the placebo arm to the active treatment arm. Patient crossover on disease progression was high in both trials (SELECT: 87.8%, DECISION: 75%) [24, 25]. In addition, in both trials, patients in either arm were also eligible to receive subsequent anti-cancer treatments that were not part of the trial protocols [24, 25]. In the SELECT trial, at the primary data-cut, 15.7% of patients randomized to lenvatinib and 12.2% of patients randomized to placebo, had received subsequent treatment (data from CSR) including treatment with another TKI (data from CSR). Of those who received subsequent treatment, 17.1% of patients in the lenvatinib arm received pazopanib and 14.6% received sorafenib (data from CSR). In the placebo arm, the respective proportions were 18.8 and 12.5% (data from CSR). In the DECISION trial, at the primary data-cut, 20.3% of patients randomized to sorafenib and 8.6% of patients randomized to placebo received subsequent treatments [27]. Information on the specific agents used during the DECISION trial follow-up period was not collected.

The median duration of follow-up at the primary data-cut was approximately 17 months in both trials [26, 27]. OS results were also reported at a second and third data-cut in both trials [24, 25]. At the third data-cut, the median length of follow-up was approximately 38 months in the SELECT trial [24] and 36 months in the sorafenib arm of the DECISION trial [25] (length of follow-up data have only been reported for the sorafenib arm of this trial).

The OS results from both trials were adjusted for treatment crossover using the Rank Preserving Structural Failure Time Model (RPSFTM) [60]. No adjustments were made, in either trial, to take into account subsequent anti-cancer treatment, as there is no recognised approach for making such adjustments.

A key difference in eligibility between the two RCTs was that the SELECT trial permitted the enrolment of patients who had been previously treated with a TKI (including sorafenib) [26], whilst patients recruited to the DECISION trial were all TKI naïve [27]. Overall, 25.3% of patients in the lenvatinib arm and 20.6% of patients in the placebo arm of the SELECT trial had received prior treatment with a TKI [26]. Approximately three quarters of patients who received a TKI in the SELECT trial had previously been treated with sorafenib (77.2% in the lenvatinib arm and 77.8% in the placebo arm) [26].

#### Characteristics of prospective observational studies and evidence reviews (supporting evidence)

All nine of the prospective observational studies were single arm studies and included patients whose disease was described as being radioactive iodine refractory [28–30, 33, 35, 36], resistant to radioactive iodine [31, 32] or who may have received multiple treatments of radioactive iodine [34]. Two studies [29, 36] investigated the efficacy and safety of lenvatinib and seven studies considered the efficacy of sorafenib [28, 30–35]; one study included no safety data [34].

Most of the observational studies were conducted in single countries (and often in single centres) in Europe [28, 31, 34, 35], the US [33, 58], and Asia [30, 36]. However, there was one multi-centre international study of lenvatinib (Study 201) [29]. Where reported, patients were recruited prior to the commencement of the SELECT [26] and DECISION [27] trials, the exception was a Japanese study of lenvatinib (Study 208) [36] that began after recruitment to the SELECT trial had ended.

The median length of follow-up, as reported in the EPAR for lenvatinib [56], was longer in the observational studies of lenvatinib [29, 36] than in the SELECT trial [24]: 40 months in Study 208 [56] and 51.6 months in Study 201 [56]. Conversely, where reported [28, 34, 35], the median length of follow-up in the observational studies of sorafenib was shorter for OS but longer for other outcomes than in the DECISION trial [25]: 19 months [34] to 25 months [35].

The number of patients included in the nine prospective observational studies varied from nine [30] to 58 [29]. In total, across all studies, 109 patients were treated with lenvatinib, of whom 83 had RR-DTC; 213 patients were treated with sorafenib, of whom 186 had RR-DTC. Other patients included in four of the studies [28, 33, 36, 58] had anaplastic (*n* = 26) or medullary (*n* = 27) carcinoma. Participant characteristics were reported for all treated patients in each study and, where reported, median age ranged from 55 years [28] to 64 years [33]. Where reported, four studies included a majority of males [28, 29, 33, 35] and three studies included a majority of females [31, 34, 58]. Only two studies explicitly stated that patients could have received a prior TKI [29, 34] and, in these studies, the proportion of patients who did receive a prior TKI ranged from 11.8% [34] to 29.3% [29].

Overall, 11 evidence reviews included evidence for lenvatinib and sorafenib [24, 25, 37–43, 46, 47]. Two reviews only included observational studies of sorafenib [44, 45].

The earliest review, which presented evidence narratively, was published in 2013 [37] and the most recent reviews (from 2017) were the evidence submissions from the sponsors of lenvatinib [24] and sorafenib [25]. Both of the evidence submissions [24, 25] included modified versions of the indirect comparisons of lenvatinib versus sorafenib originally conducted by Tremblay et al. 2016 [46]; the original results [46] were also reported in the Canadian Agency for Drugs and Technologies in Health (CADTH) submission for lenvatinib [39]. One other publication [42], included an indirect comparison of lenvatinib versus sorafenib. The two reviews that included only observational studies of sorafenib meta-analyzed the data from the studies they included [44, 45].

### Quality assessment of included studies

Overall, the risk of bias was considered to be low in both RCTs (Additional file 2: Online Resource 2). The quality of nine of the evidence reviews [24, 25, 37–39, 42–45] was considered to be good (Additional file 3: Online Resource 3).

### Results from the included studies

#### Primary evidence efficacy evidence

We have reported RCT evidence from the primary data-cuts of the SELECT and DECISION trials [26, 27], with the exception of OS data, which are reported for the third data-cut [24, 25]. The results for OS, PFS and ORR from the RCTs are summarized in Table 2.

**Table 2:** Summary of efficacy findings from the SELECT and DECISION trials

Outcome	SELECT trial		DECISION trial	
	Lenvatinib *N* = 261	Placebo *N* = 131	Sorafenib *N* = 207	Placebo *N* = 210
OS^a^				
Median, months	41.6	34.5	39.4	42.8
(95% CI)	(31.2-NE)	(21.7-NE)	(32.7–51.4)	(34.7–52.6)
Unadjusted HR (95% CI)	0.84 (0.62–1.13)		0.92 (0.71–1.21)	
RPSFTM adjusted OS HR	0.54		0.77	
(95% CI) ^b^	(0.36–0.80)		(0.42–1.79)	
PFS^c^				
Median, months	18.3	3.6	10.8	5.8
(95% CI)	(15.1-NE)	(2.2–3.7)	(CIs NR)	(CIs NR)
Stratified HR (95% CI)	0.21 (0.14–0.31)		0.59 (0.45–0.76)	
Objective tumour response				
rate^c, d^ (%)	64.8	1.5	12.2	0.5
(95% CI)	(59–70.5)	(0–3.6)	(8–17.7)	(0–2.7)
Odds Ratio (95% CI)	28.87 (12.46–66.86)		NR	
*P* value	*p* < 0.0001		*p* < 0.0001	

*CI* Confidence interval, *HR* Hazard ratio, *IPE* Iterative Parameter Estimation, *NE* Not estimable, *NR* Not reported, *OS* Overall survival, *PFS* Progression-free survival, *RPSFTM* Rank Preserving Structural Failure Time Model

^a^Data from final data-cut

^b^Bootstrapping CIs

^c^Assessed by blinded independent review at primary data-cut

^d^Unlike the SELECT trial, patients who were unevaluable for response were excluded from the analyses in the DECISION trial. There were 18 (4.3%) patients who were excluded from the objective tumour response analyses in the DECISION trial, 9 (4.3%) patients in each arm [27]

Source: [26, 27] with additional OS data from Eisai Ltd. 2017 [24] and Bayer HealthCare 2017 [25] and additional ORR data (95% CIs) from European public assessment report (EPAR) for lenvatinib [51] and EPAR for sorafenib [56]

For OS, no statistically significant differences between trial arms were found in either trial [24, 25]. When OS results from both trials were adjusted for treatment crossover, the difference was reported to be statistically significant in the SELECT trial, favouring lenvatinib over placebo [24] but a similar finding was not reported in the DECISION trial for sorafenib versus placebo [25]. Compared to placebo, median PFS and ORR were improved with lenvatinib in the SELECT trial [26] and with sorafenib in the DECISION trial [27]. The difference in ORR between trial arms was particularly pronounced in the SELECT trial, difference in ORR 63.2% (95% CI: 57.1 to 69.4%) [26]; the difference in ORR in the DECISION trial was 11.7% (95% CI: 7.0 to 16.5%). Differences between arms were reported to be statistically significant for PFS and ORR in both trials [26, 27].

As some patients in the SELECT trial had previously received a TKI (including sorafenib), subgroup analyses were conducted to assess the effect of this previous treatment and the results have been reported for median PFS and ORR [26]. Median PFS was longer for patients treated with lenvatinib compared with placebo, irrespective of whether patients had received a TKI [26]. Median PFS for those previously treated was 15.1 versus 3.1 months (HR 0.22, 95% % confidence interval [CI]: 0.12 to 0.41) and for TKI-naïve patients median PFS was 18.7 versus 3.6 months (HR 0.20, 95%CI CI: 0.14 to 0.27) [26]. Similarly, ORR was improved for patients treated with lenvatinib whether or not they had been previously treated with a TKI (62.1% versus 3.7%; odds ratio [OR] 15.57, 95% CI: 4.06 to 59.72), or not (65.6% versus 1.0%; OR 58.88, 95% CI: 18.95 to 182.91) [26].

#### Indirect comparison of lenvatinib versus sorafenib

In the absence of direct clinical trial evidence comparing treatment with lenvatinib versus treatment with sorafenib, we assessed the feasibility of conducting an indirect comparison to obtain estimates of the relative efficacy and safety of these two treatments. As both the SELECT and DECISION trials shared a common comparator (placebo), it is possible to construct a network. Indeed, indirect comparisons have been reported in evidence reviews [24, 25, 39, 42, 46]. For an indirect comparison to be reliable: (i) trial and participant characteristics must be sufficiently similar (ii) survival hazard profiles for the shared comparator should be similar and (iii) within trials, hazards should be proportional (since Cox proportional hazard [PH] modelling [61] was used to generate OS, RPSFTM-adjusted OS and PFS hazard ratios [HRs]). We therefore tested whether all these assumptions were supported by the data.

**Fig. 2:**
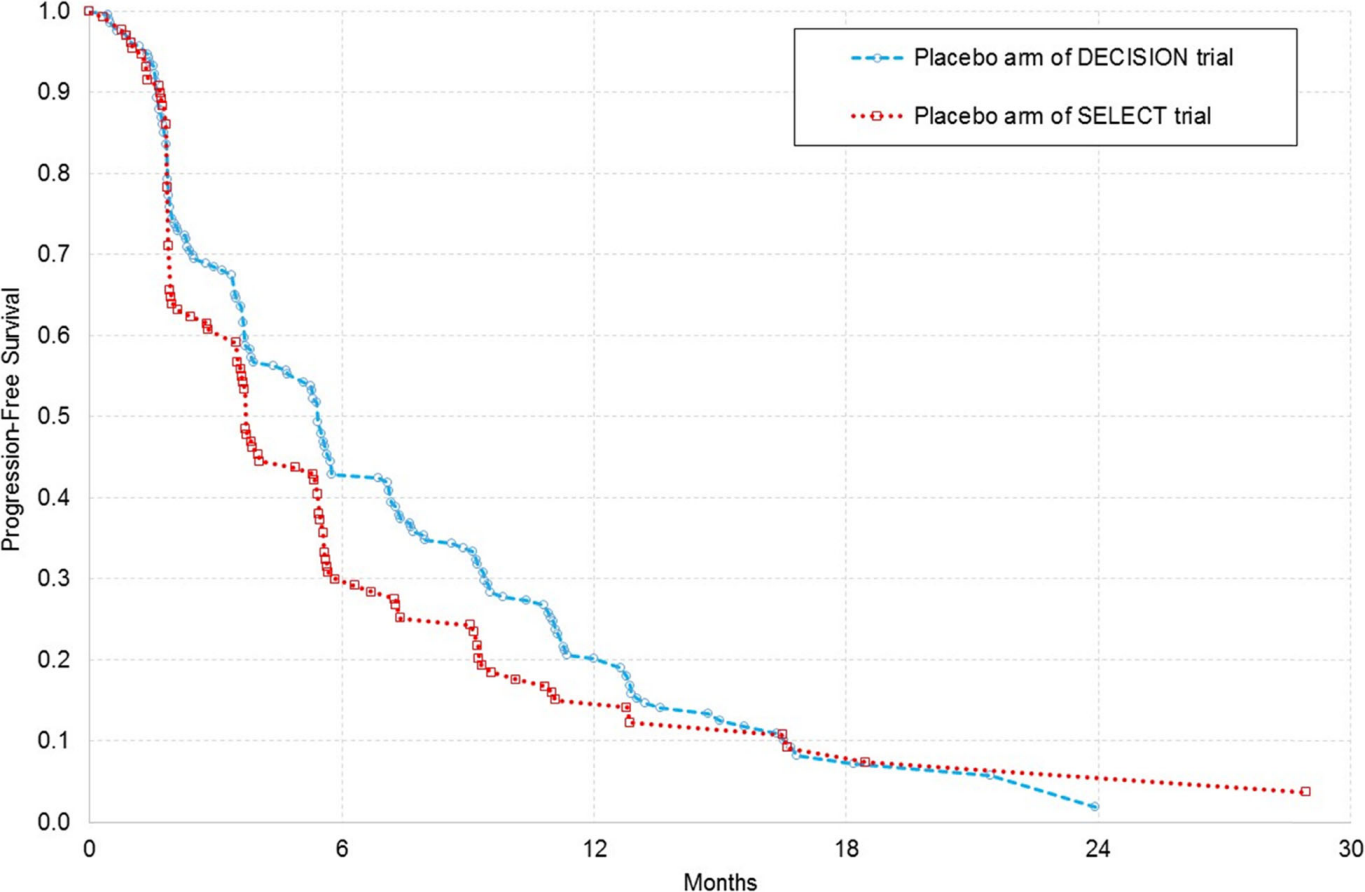
Comparison of progression-free survival in the placebo arms of the DECISION and SELECT clinical trials. Source: Data provided during the NICE appraisal by Eisai Ltd. and Bayer HealthCare

In relation to (i), we found that there were a number of differences in trial and participant characteristics, which were most pronounced when comparing the placebo arms of the two trials, as highlighted in Table 3. In relation to (ii), from an examination of PFS data, it was also evident that the survival risk profiles of the shared comparator (the placebo arms) were not comparable (Fig. 2). In relation to (iii), we tested the validity of the proportional hazards assumption for OS, RPSFTM-adjusted OS and PFS against a non-linear (quadratic) counterfactual using an analysis of variance (ANOVA) test. With the exception of unadjusted OS data in the DECISION trial, we found the PH assumption was violated and thus the network of evidence was compromised for all efficacy outcomes. Therefore, we did not undertake an indirect comparison to compare the efficacy of lenvatinib versus sorafenib.

**Table 3:**
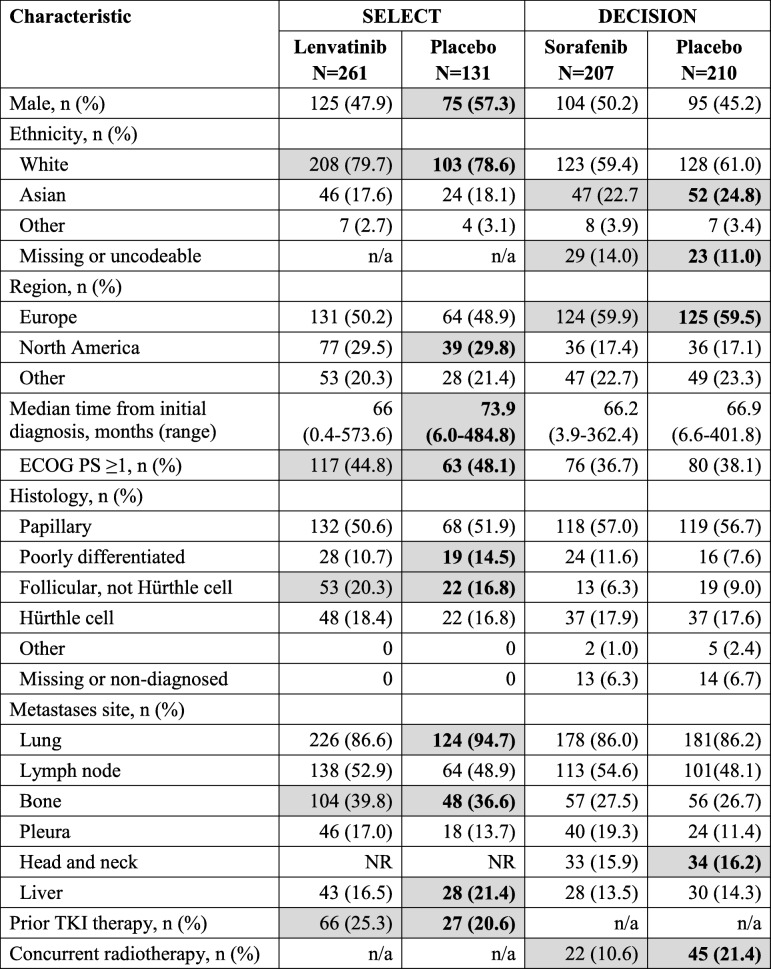
Differences in characteristics of the SELECT and DECISION trials (bold text/shaded cells)

*DTC* Differentiated thyroid cancer, *ECOG* Eastern Cooperative Oncology Group, *n/a* Not applicable, *NR* Not reported, *PS* Performance status, *TKI* Tyrosine kinase inhibitor

Sources: Eisai Ltd. 2017 [24, 26], EPAR for lenvatinib [27, 51] and appendix to Bayer HealthCare 2017 [25]

Text in bold relates to the most notable differences between placebo arms and shaded cells the most notable differences between trials in any arm

#### Supporting efficacy evidence

Efficacy findings from the observational studies [28–31, 33–36, 59], and meta-analyses conducted by the authors of two sorafenib reviews [44, 45] are summarised in Table 4. Data were also extracted from the EPAR for sorafenib [56] for OS and ORR for one of the observational studies [33] and for ORR for another observational study [28]. This is because these results were not presented only for patients with RR-DTC in the published papers of these studies.

**Table 4:** Summary of efficacy data from observational studies and meta-analyses

Outcome, months	Lenvatinib	Sorafenib		
	Range from observational studies	Range from observational studies	Estimate from meta-analysis by Thomas et al. 2014 [45]	Estimate from meta-analysis by Shen et al. 2014 [44]
OS, median	31.8–32.3 ^[2]^	23–34.5 ^[3] a^	- ^c^	- ^c^
PFS, median	12.6–25.8 ^[2]^	12–22.1 ^[4] b^	17.9	- ^c^
95% CI			17.9–18 ^[7]^	
ORR, %	50–68 ^[2]^	15–38.3 ^[7]^	20.9	22
95% CI			14.3–27.5 ^[6]^	15–28 ^[7]^

- = not applicable, *CI* Confidence interval, *ORR* Objective tumour response rate, *OS* Overall survival, *PFS* Progression-free survival

^a^An additional study reported that the median OS had not been met [28]

^b^One other study reported that the median PFS had not been met [28] and another reported mean PFS only (9.7 months) [30]; in this latter study sorafenib was studied at half the dose of all other studies and included only 9 patients

^c^ No meta-analyses were identified

^[x]^ denotes the number of studies from which data are derived

Median OS reported in both observational studies of lenvatinib [29, 36] was approximately 32 months, lower than the median OS estimates reported for both arms of the SELECT trial (lenvatinib: 41.6 months, placebo: 34.5 months) [24]. Similarly, median OS reported in three studies of sorafenib [33, 35, 59], which ranged from 23 months [33] to 34.5 months [35], was lower than median OS reported in either arm of the DECISION trial (sorafenib: 39.4 months, placebo: 42.8 months) [25]. Median OS could not be estimated in one other study of sorafenib, as it had not yet been reached [28].

Median PFS and ORR for patients treated with lenvatinib were lower in one study (median PFS: 12.6 months, ORR: 50%) [29] and higher in another (median PFS: 25.8 months, ORR: 68%) [36] than reported for patients treated with lenvatinib in the SELECT trial (median PFS: 18.3 months, ORR: 64.8%) [26]. Median PFS was higher in all four prospective observational studies that reported a median [33–35] as was ORR in all prospective observational studies [28, 30, 31, 33–35, 59] than in the DECISION trial [27]; range of median PFS was 12 months [34] to 22.1 months [59] in the observational studies and 10.8 months in the sorafenib arm of the DECISION trial [27], range of ORRs was 15% [33] to 38.3% [59] in the observational studies and 12.2% in the sorafenib arm of the DECISION trial [27]. Reflecting these findings, authors of the sorafenib meta-analyses of single-arm studies [44, 45] reported a higher median PFS and ORR than reported for patients treated with sorafenib in the DECISION trial [27]; median PFS of 17.9 months [45] and ORR of 21 to 22% [44, 45] in the meta-analyses.

Two published papers have reported efficacy results from indirect comparisons of lenvatinib with sorafenib [42, 46] utilising data from the SELECT and DECISION trials [26, 27]. There were no statistically significant differences in OS (whether RPSFTM-adjusted, or not) but in both papers, it was reported that PFS was significantly better with lenvatinib versus sorafenib (HR 0.36, 95% CI: 0.22 to 0.57) [42, 46]. The results from a matched adjusted indirect comparison (MAIC) for OS and PFS were very similar to the unmatched results [46]. One of the published papers also included a comparison for ORR and found no statistical significance between lenvatinib and sorafenib (relative benefit 1.72, 95% CI: 0.15 to 19.40) [42].

#### Primary safety evidence

Safety evidence from the SELECT and DECISION trials is summarised in Table 5. The majority of AE data for the SELECT trial is taken from the Eisai Ltd. evidence submission [24] as, similar to the reporting in the DECISION trial [27], this reported treatment-emergent AEs, whereas the primary published paper mostly reported treatment-related AEs [26]. Treatment with both lenvatinib and sorafenib led to an increase in the incidence of AEs versus treatment with placebo [24, 27]. Dose interruptions and reductions were very frequent for patients treated with both lenvatinib and sorafenib [26, 27]. Fatal AEs were recorded for 7.7% of patients treated with lenvatinib and 4.6% of patients who received placebo in the SELECT trial [26]. Fatal AEs in the DECISION trial were recorded for 5.8% of patients treated with sorafenib and 2.9% of patients in the placebo arm [27].

**Table 5:** Summary of safety data in the SELECT and DECISION trials

Outcome, n (%)	SELECT trial		DECISION trial	
	Lenvatinib *N* = 261	Placebo *N* = 131	Sorafenib *N* = 207	Placebo *N* = 209
Any adverse event	260 (99.6)	118 (90.1)	204 (98.6)	183 (87.6)
Any Grade ≥ 3 adverse event	223 (85.4)	39 (29.8)	133 (64.3)	63 (30.1)
Most common all-Grade AEs^a^				
Hypertension	181 (69.3)	19 (14.5)	84 (40.6)	26 (12.4)
Diarrhoea	173 (66.3)	22 (16.8)	142 (68.6)	32 (15.3)
Decreased appetite / anorexia	139 (53.3)	24 (18.3)	66 (31.9)	10 (4.8)
Weight loss	132 (50.6)	19 (14.5)	97 (46.9)	29 (13.9)
Nausea	121 (46.4)	33 (25.2)	43 (20.8)	24 (11.5)
Fatigue	110 (42.1)	32 (24.4)	103 (49.8)	53 (25.4)
Hand-foot syndrome	84 (32.2)	1 (0.8)	158 (76.3)	20 (9.6)
Rash or desquamation	48 (18.4)	2 (1.5)	104 (50.2)	24 (11.5)
Alopecia	32 (12.3)	7 (5.3)	139 (67.1)	16 (7.7)
Most common Grade ≥ 3 AEs^b^				
Hypertension	112 (42.9)	5 (3.8)	20 (9.7)	5 (2.4)
Hand-foot syndrome	9 (3.4)	0	42 (20.3)	0
Weight loss	31 (11.9)	1 (0.8)	12 (5.8)	2 (1)
Proteinuria	26 (10)	0	0	0
Treatment interruptions, reductions or discontinuations because of an adverse event				
Dose interruptions	215 (82.4)	24 (18.3)	137 (66.2)	54 (25.8)
Dose reductions	177 (67.8)	6 (4.6)	133 (64.3)	19 (9.1)
Discontinued treatment	43 (16.5)	6 (4.6)	39 (18.8)	8 (3.8)

^a^ ≥ 40% in any arm

^b^ ≥ 10% in any arm

Source: Eisai Ltd. 2017 [24, 26, 27] and clinical study report for the DECISION trial (unpublished)

The most frequently reported AEs occurring in around two-thirds of patients were, for lenvatinib, hypertension and diarrhoea [24] and, for sorafenib, hand-foot syndrome, diarrhoea and alopecia [27]. Hypertension was a very frequent Grade ≥ 3 AE reported with lenvatinib [24] and hand-foot syndrome was a frequent Grade ≥ 3 AE reported with sorafenib [27].

Analyses have been undertaken to determine the median time to onset of five AEs for patients treated with lenvatinib in the SELECT trial [48], and eight AEs with for patients treated with sorafenib in the DECISION trial [52]. The results suggest that, when treated with either lenvatinib or sorafenib, most AEs typically occur early, with a decrease in incidence, prevalence and severity over time [48, 52]. However, hypertension was a notable AE omitted from the analysis of lenvatinib data [48].

The incidences of any all-Grade and Grade ≥ 3 AEs for patients treated with lenvatinib were similar in patients who had received a prior TKI to those who had not [49, 50]. The proportion of patients who had at least one lenvatinib dose reduction was also similar between these two subgroups [49, 50].

#### Supporting safety evidence

The safety data from the prospective observational studies of lenvatinib [29, 36], prospective observational studies of sorafenib [28, 33, 35, 57] and meta-analyses of observational studies of sorafenib [44, 45] are summarised in Table 6. Prospective observational study authors report either treatment-emergent [28, 29, 35, 36] or treatment-related AEs [33, 57]. The meta-analyses appear to include a combination of treatment-emergent and treatment-related AEs [44, 45].

**Table 6:** Summary of safety data in the observational studies and meta-analyses

Event	Lenvatinib TEAEs, range from studies	Sorafenib TEAEs, range from studies	Sorafenib TRAEs, range from studies	Sorafenib AEs	
				Median (95% CI) from meta-analysis by Thomas et al. 2014^a^	Median (95% CI) from meta-analysis by Shen et al. 2014
Most common all-Grade AEs (%)^b^					
Hypertension	76–90 ^[2]^	21–42 ^[3]^	43 ^[2]^	36 (27–46)^[7]^	52 (33–72)^[7]^
Diarrhoea	55–67 ^[2]^	52–77 ^[3]^	75–80 ^[2]^	70 (62–78)^[7]^	68 (59–77)^[7]^
Decreased appetite	52–78 ^[2]^	29 ^[1]^	20–82 ^[2]^	–	–
Weight loss	69 ^[1]^	29–58 ^[2]^	60–82 ^[2]^	57 (39–75)^[7]^	52 (33–72)^[7]^
Nausea	50 ^[1]^	10–27 ^[2]^	30–55 ^[2]^	–	–
Fatigue	60–73 ^[2]^	59 ^[1]^	63–66 ^[2]^	–	–
Hand foot syndrome	22–77 ^[2]^	71–79 ^[3]^	63–91 ^[2]^	74 (64–83)^[7]^	80 (68–91)^[7]^
Rash	24 ^[1]^	55–88 ^[2]^	79–85 ^[2]^	67 (52–82)^[7]^	66 (50–82)^[7]^
Alopecia	9 ^[1]^	52–74 ^[2]^	43–79 ^[2]^	–	–
Proteinuria	61–64 ^[2]^	–	–	–	–
Stomatitis/ mucositis	31–57 ^[2]^	27–48 ^[3]^	16–47 ^[2]^	–	–
Cough	45 ^[1]^	–	–	–	–
Headache	43 ^[1]^	15 ^[1]^	16 ^[1]^	–	–
Dysphonia	43 ^[1]^	–	–	–	–
Infection	–	68 ^[1]^	–	–	–
Hypocalcaemia	–	48 ^[1]^	–	–	–
Dry skin	–	–	84 ^[1]^	–	–
Pruritis	–	–	77 ^[1]^	–	–
Flatulence	–	–	70 ^[1]^	–	–
Abdominal/ rectal pain	–	–	68 ^[1]^	–	–
Arthralgia	–	–	61 ^[1]^	–	–
Most common Grade ≥ 3 AEs (%)^c^					
Hypertension	10 ^[1]^	6–16 ^[2]^	4–13 ^[2]^	7 (3–12)^[7]^	–
Hand foot syndrome	2 ^[1]^	23–44 ^[2]^	7 ^[2]^	19 (8–31)^[7]^	–
Weight loss	12 ^[1]^	0–10 ^[2]^	5–10 ^[2]^	5 (1.2–9)^[7]^	–
Proteinuria	10 ^[1]^	–	–	–	–
Diarrhoea	10 ^[1]^	3–7 ^[2]^	4–7 ^[2]^	7 (3–10)^[7]^	–
Fatigue	9 ^[1]^	9 ^[2]^	16 ^[1]^	10 (4–16)^[7]^	–
Stomatitis/ mucositis	2 ^[1]^	9–10 ^[2]^	0–2	4 (1–7)^[7]^	–
Rash	0 ^[1]^	6–16 ^[2]^	4–18 ^[2]^	7 (3–11)^[7]^	–
Myocardial infarction	–	10 ^[1]^	–	–	–
Hand or foot pain	–	–	12 ^[1]^	–	–
Arthralgia	–	–	11 ^[1]^	–	–
Treatment interruptions, reductions or discontinuations because of an adverse event (%)					
Dose interruptions	74 ^[1]^	82 ^[1]^	–	–	–
Dose reductions	66 ^[1]^	42–100 ^[2]^	47–55 ^[2]^	–	–
Discontinued treatment	2–26 ^[2]^	23 ^[1]^	20 ^[1]^	–	–

- = not reported or not applicable; *AE* Adverse event, *C* Confidence interval, *TEAE* treatment-emergent adverse event; *TRAE* Treatment-related adverse event

^a^One of the included studies did not include only patients with RR-DTC

^b^ ≥ 40% in any study

^c^ ≥ 10% in any study

^[x]^ denotes the number of studies from which data are derived

Although there were differences in the incidences of some AEs across studies [28, 29, 33, 35, 36, 44, 45, 57] and compared to the SELECT and DECISION trials [24, 27], the most common types of AEs with both drugs were similar to those found in the RCTs. As with the RCT evidence [26, 27], dose interruptions and reductions were very frequent for patients treated with either lenvatinib [29] or sorafenib [35, 57].

One of the published reviews [42] compared the relative risk of AEs from treatment with lenvatinib with treatment from sorafenib via an indirect comparison utilising data from the SELECT and DECISION trials [26, 27]. The authors reported that the risk of all-Grade AEs was similar (OR 2.55, 95% CI: 0.59 to 11.57) [42]. The authors also tested for differences for 17 different types of AEs (treatment-related for lenvatinib and treatment emergent for sorafenib) and found that compared with sorafenib, lenvatinib significantly increased the risk of hypertension (risk ratio [RR] 2.31, 95% CI: 1.18 to 4.53) but significantly reduced the risk of alopecia (RR 0.33, 95% CI: 0.12 to 0.94) [42]. There were no significant differences for the other 15 AEs, which included the other most common AEs reported in the SELECT and DECISION trials [26, 27].

The authors of one of the indirect comparisons also presented results for serious AEs (SAEs), serious treatment-related AEs and treatment discontinuation due to AEs [42]. The only significant difference was that lenvatinib increased the risk of serious treatment-related AEs compared to sorafenib (RR 4.02, 95% CI: 1.69 to 9.6) [42].

#### Evidence for health-related quality of life with treatment

HRQoL data were only collected during the DECISION trial and the results were presented in a conference abstract [55] and in Bayer HealthCare’s evidence submission to NICE [25]. Cancer-specific HRQoL was measured using the Functional Assessment of Cancer Therapy - General (FACT-G) questionnaire [62] and general health status was measured using the generic EuroQol five dimensions, three-level questionnaire (EQ-5D-3 L) and the EQ-5D visual analogue scale (VAS) [63]. All questionnaires were self-administered at baseline and day 1 of every 28-day cycle until disease progression [55]. The overall questionnaire completion rate during the DECISION trial was reported to be > 96% [25].

At baseline, patients’ HRQoL data were considered by the authors to be comparable to a normative adult cancer population [25, 55]. However, at the first assessment (cycle 2, day 1), HRQoL scores (FACT-G, EQ-5D-3 L and VAS) had deteriorated in the sorafenib arm [25, 55]. Thereafter, the sorafenib arm scores remained similar to the scores recorded at the first assessment until disease progression [25, 55]. Scores for the placebo arm remained very similar to the baseline scores at the first assessment and all subsequent assessments until disease progression [25, 55]. Results from a mixed linear model showed that, compared with placebo, the FACT-G score was 3.45 points lower in the sorafenib arm than the placebo arm (*p* = 0.0006) [25, 55]. This is reported to represent a clinically meaningful difference between arms in favour of the placebo arm [25, 55]. While the between arm differences were statistically significant for both EQ-5D-3 L and VAS (*p* < 0.0001), the treatment effects (− 0.07 and − 6.75, respectively) were reported to be of a small magnitude which did not reach the threshold considered to represent a clinically meaningful difference [25, 55].

## Discussion

The aim of this review was to compare the clinical effectiveness evidence for lenvatinib or sorafenib in relation to BSC and also to compare the effectiveness of both drugs with each other.

Trial results show that both drugs are more efficacious in terms of median PFS [26, 27] and ORR [26, 27] but also result in more AEs than placebo [24, 27]. Placebo can be considered to be a proxy for BSC in both trials, even though concurrent use of palliative radiotherapy was not permitted for patients in the SELECT trial (data from CSR). Some of the most common types of AEs differ by drug, most notably hypertension being very common with lenvatinib [24] and hand-foot syndrome being very common with sorafenib [27]. We were unable to determine the true impact of lenvatinib and sorafenib on OS or how both drugs, particularly lenvatinib, impact upon HRQoL. This is because OS is confounded by treatment crossover in both trials [26, 27] and HRQoL data is limited to reports of sorafenib from the DECISION trial [25, 55].

It should however be noted that results for OS (except in the case of the DECISION trial), RPSFTM-adjusted OS and PFS described as statistically significant (or otherwise) should be interpreted with caution, since we found for that for these outcomes, the PH assumption was violated. It is therefore not possible to ascertain whether the HRs are overestimates or underestimates of the effect of the intervention versus placebo in either trial.

In conducting a feasibility assessment of performing indirect comparisons, we identified potential differences in trial and population characteristics at baseline. Since the PH assumption for OS and PFS data were also found to be violated, we considered that the validity of conducting an indirect comparison (matched or otherwise) using standard methods was questionable. Importantly, we also identified differences in the survival risk profiles of patients in the placebo arms of the trials. These differences may reflect known or unknown differences in trial and participant characteristics. The identification of these differences was our primary reason for considering an indirect comparison to be inappropriate. Of note, the CADTH have also considered the populations to be different, stating that the SELECT trial population had more aggressive disease as reflected by PFS in the placebo arms [39]. Furthermore, in its consideration of the evidence base during the MTA process, the NICE Appraisal Committee agreed that the Kaplan-Meier plots for PFS in the placebo arms of the trials were sufficiently different to suggest there were important differences limiting the robustness of the indirect comparisons [64].

NICE guidance is based on the recommendations of the NICE Appraisal Committee. The extent to which the findings from either of the SELECT and DECISION trials are generalizable to clinical practice was one of the key considerations for the NICE Appraisal Committee [64]. In clinical practice, patients are often not treated with lenvatinib or sorafenib unless their disease is symptomatic, or they have clinically significant progressive disease (e.g. obvious radiological or biochemical progression). Data published in the EPAR for sorafenib [56] indicate that approximately 20% of patients in the DECISION trial had been retrospectively defined as being symptomatic; the equivalent proportion in the SELECT trial was unknown. To be eligible for entry into both trials, patients were required to have had radiographic evidence of disease progression within the last 12 months (SELECT trial) or 14 months (DECISION trial) [26, 27]. Arguably these eligibility criteria suggest that patients had clinically significant disease that was likely to be rapidly progressing, if left untreated. Indeed, clinical opinion presented to the NICE Appraisal Committee was that if patients were not yet symptomatic in the trials, it was likely they would soon become symptomatic [64]. The evidence from both trials, even though it appears to include slightly different trial populations, was, therefore, considered to be generalizable to clinical practice.

In the absence of results from reliable indirect comparisons, findings from observational studies provide important supporting evidence. The magnitude of effects in relation to OS, PFS and the incidence of some AEs differed in prospective observational studies [28–31, 33–36, 57, 59] and meta-analyses [44, 45] to the RCT findings [24–27]. There are a number of reasons that could explain this. First, as with the RCTs, differences in unknown patient characteristics may be contributory factors. Second, the differing lengths of follow-up should be considered. Third, all of the prospective observational studies were relatively small, and so the results are more prone to being influenced by any outlying cases. However, while caution needs to be exercised in comparing results across studies of different study populations, the combined evidence from RCTs [26, 27] and observational studies [28–31, 33–36, 59] suggests ORR may be higher for patients treated with lenvatinib than for patients treated with sorafenib. Evidence from observational studies [28–31, 33, 35, 36, 57] and meta-analyses [44, 45] also show that many common AEs reported with lenvatinib and sorafenib in the RCTs [26, 27] are also experienced by patients treated with these drugs in other study populations. The evidence shows that some AEs are very common to both lenvatinib and sorafenib (e.g. diarrhoea), whereas other AEs tend to be more drug specific (e.g. hypertension with lenvatinib and hand-foot syndrome with sorafenib) [28, 29, 33, 35, 36, 44, 45, 57]. Therefore, the body of evidence taken as a whole supports the NCCN recommendation that “The decision of whether to use lenvatinib (preferred) or sorafenib should be individualized for each patient based on likelihood of response and comorbidities” [10].

No HRQoL data for lenvatinib are available from either the SELECT trial or the supporting observational studies, [29, 36]. Only the DECISION trial collected HRQoL data for patients treated with sorafenib, and then only until the end of treatment [25, 55]. In the DECISION trial, “mild” reductions in HRQoL were reported for patients treated with sorafenib compared to those receiving the placebo [25, 55]. Given the different objective tumour response rates and types of AEs reported in the studies of lenvatinib, HRQoL data for patients treated with lenvatinib would have been very informative. It is unclear whether, for patients treated with lenvatinib, obtaining an objective response to treatment is associated with improved HRQoL, or if they too would experience “mild” reductions in HRQoL. The exploration of HRQoL associated with treatment with both drugs is an area requiring further research.

Another area where further research is required relates to the sequential use of lenvatinib and sorafenib. Subgroup analysis results from the SELECT trial suggest that differences in PFS, ORR and AEs for lenvatinib versus placebo were similar regardless of whether a patient had been previously treated with a TKI, or not [26, 49, 50]. However, no OS evidence has been reported for these subgroups. Furthermore, the number of patients in these subgroups, particularly in the placebo arm, is small. Importantly, there is no evidence for the efficacy or safety of treatment with sorafenib following treatment with lenvatinib.

The evidence presented in our review has been used as the basis for making recommendations for practice in England. Guidance was issued by NICE in August 2018 [64]. In drafting the guidance, the NICE Appraisal Committee considered the uncertainties identified in our review, alongside cost effectiveness evidence, and testimonies from clinical and patient experts. NICE guidance recommends the use of lenvatinib or sorafenib for treating RR-DTC if both drugs are provided at a discounted price [64]. However, NICE guidance also includes the restriction that lenvatinib or sorafenib are only available to patients who have not previously received treatment with a TKI or “if they have had to stop taking a TKI within 3 months of starting it because of toxicity (specifically, toxicity that cannot be managed by dose delay or dose modification)” [64]. The reason given for this restriction is because NICE considered that there is “not enough clinical evidence and no cost-effectiveness evidence to determine whether the treatments are effective when used sequentially” [64]. Restricted use of lenvatinib or sorafenib differs to the licensing [16–19] and also reimbursement approval received elsewhere in the UK [21].

## Conclusions

It is not possible to reliably estimate the relative effectiveness of lenvatinib versus sorafenib for treating RR-DTC, but the evidence base clearly demonstrates improvements in PFS and ORR for these treatments when compared with placebo, a proxy for BSC. The improvements in PFS and ORR are, however, accompanied by an increased risk of AEs, whilst the effect on patients’ OS and HRQoL remains uncertain. Given the slightly different safety profiles of lenvatinib and sorafenib, the evidence from our review supports clinical guideline recommendations that the choice of treatment should consider each patient’s circumstances, including their need for a response to treatment and comorbidities.

## Supplementary information

**Additional file 1.** Online Resource 1. Search strategies for each electronic database used to identify studies.

**Additional file 2.** Online Resource 2. Risk of bias assessment of the SELECT and DECISION trials.

**Additional file 3.** Online Resource 3. Quality assessment of systematic review evidence included.

## Data Availability

Additional information relating to the search strategies to identify studies and quality assessment of included studies is provided in the supplementary information files (Additional file 1, Additional file 2 and Additional file 3).

## Abbreviations

AEs: Adverse events
ANOVA: Analysis of variance
ATA: American Thyroid Association
BSC: Best supportive care
CADTH: Canadian Agency for Drugs and Technologies in Health
CI: Confidence interval
CRD: Centre for Reviews and Dissemination
CSR: Clinical study report
DTC: Differentiated thyroid cancer
EPAR: European public assessment report
EQ-5D VAS: EuroQol five dimensions visual analogue scale
EQ-5D-3 L: EuroQol five dimensions, three-level questionnaire
EU: European Union
FACT-G: Functional Assessment of Cancer Therapy – General
FTC: Follicular carcinoma
HRQoL: Health-related quality of life
LRiG: Liverpool Reviews and Implementation Group
MTA: Multiple technology appraisal
NCCN: National Comprehensive Cancer Network
NHS: National Health Service
NICE: National Institute for Health and Care Excellence
OR: Odds ratio
ORR: Objective tumour response rate
OS: Overall survival
PFS: Progression-free survival
PH: Proportional hazard
PRISMA: Preferred Reporting Items for Systematic Reviews and Meta-Analyses
PTC: Papillary carcinoma
RCT: Randomized controlled trial
RPSFTM: Rank Preserving Structural Failure Time Model
RR: Risk ratio
RR-DTC: Radioactive iodine refractory differentiated thyroid cancer
TKI: Tyrosine kinase inhibitor
TSH: Thyroid stimulating hormone
UK: United Kingdom
US: United States
