# Supplementary material for: A systematic review of lenvatinib and sorafenib for treating progressive, locally advanced or metastatic, differentiated thyroid cancer after treatment with radioactive iodine
Source: BMC Cancer. 2019 Dec 12;19:1209. doi: 10.1186/s12885-019-6369-7 (PMC6909631; doi:10.1186/s12885-019-6369-7)
Supplement: Supplementary file 1 — Additional file 1. Online Resource 1. Search strategies for each electronic database used to identify studies. [file 12885_2019_6369_MOESM1_ESM.docx]

## Online Resource 1. Search strategies

## Embase

| 1 | exp Thyroid Neoplasms/ |
| --- | --- |
| 2 | ((thyroid* or papillar* or follicular*) adj4 (Neoplasm* or Cancer* or Carcinoma* or Adenocarcinom* or Tumour* or Tumor* or Malignan* or Lump* or adenoma*)).tw. |
| 3 | (DTC or FTC or PTC).tw. |
| 4 | adenocarcinoma, follicular/ or carcinoma, papillary, follicular/ or adenocarcinoma, papillary/ |
| 5 | 1 or 2 or 3 or 4 |
| 6 | (Lenvatinib or Lenvima or E7080).tw. |
| 7 | (Nexavar or Sorafenib or bay439006).tw. |
| 8 | lenvatinib/ |
| 9 | sorafenib/ |
| 10 | 6 or 7 or 8 or 9 |
| 11 | 5 and 10 |
| 12 | limit 11 to yr="1999 -Current" |

## MEDLINE

| 1 | exp Thyroid Neoplasms/ |
| --- | --- |
| 2 | ((thyroid* or papillar* or follicular*) adj4 (Neoplasm* or Cancer* or Carcinoma* or Adenocarcinom* or Tumour* or Tumor* or Malignan* or Lump* or adenoma*)).tw. |
| 3 | (DTC or FTC or PTC).tw. |
| 4 | adenocarcinoma, follicular/ or carcinoma, papillary, follicular/ or adenocarcinoma, papillary/ |
| 5 | 1 or 2 or 3 or 4 |
| 6 | (Lenvatinib or Lenvima or E7080).tw. |
| 7 | (Nexavar or Sorafenib or bay439006).tw. |
| 8 | 6 or 7 |
| 9 | 5 and 8 |
| 10 | limit 9 to yr="1999 -Current" |

## PubMed

| #1 | Search (((thyroid* or papillar* or follicular*))) AND ((Neoplasm* or Cancer* or Carcinoma* or Adenocarcinom* or Tumour* or Tumor* or Malignan* or Lump* or adenoma*)) |
| --- | --- |
| #2 | Search (DTC or FTC or PTC) |
| #3 | Search (#1 or #2) |
| #4 | Search (Lenvatinib or Lenvima or E7080 or Nexavar or Sorafenib or bay439006) |
| #5 | Search (#3 and #4) |
| #6 | Search ("2016/07/01"[Date - Entrez] : "3000"[Date - Entrez]) |
| #7 | Search (#5 and #6) |

## Cochrane Library (CDSR/Central/ DARE/HTA)*

| #1 | MeSH descriptor: [Thyroid Neoplasms] explode all trees |
| --- | --- |
| #2 | ((thyroid* or papillar* or follicular*) near/4 (Neoplasm* or Cancer* or Carcinoma* or Adenocarcinom* or Tumour* or Tumor* or Malignan* or Lump* or adenoma*)) |
| #3 | (DTC or FTC or PTC) |
| #4 | MeSH descriptor: [Adenocarcinoma, Follicular] explode all trees |
| #5 | MeSH descriptor: [Carcinoma, Papillary, Follicular] explode all trees |
| #6 | MeSH descriptor: [Adenocarcinoma, Papillary] explode all trees |
| #7 | #1 or #2 or #3 or #4 or #5 or #6 |
| #8 | (Lenvatinib or Lenvima or E7080) |
| #9 | (Nexavar or Sorafenib or bay439006) |
| #10 | #8 or #9 |
| #11 | #7 and #10 Publication Year from 1999 to 2017 |

*CDSR=Cochrane Database of Systematic Reviews; CENTRAL=Cochrane Central Register of Controlled Trials; DARE=Database of Abstracts of Reviews of Effects; HTA=Health Technology Assessment Database
