# Supplementary material for: A systematic review of lenvatinib and sorafenib for treating progressive, locally advanced or metastatic, differentiated thyroid cancer after treatment with radioactive iodine
Source: BMC Cancer. 2019 Dec 12;19:1209. doi: 10.1186/s12885-019-6369-7 (PMC6909631; doi:10.1186/s12885-019-6369-7)
Supplement: Supplementary file 2 — Additional file 2. Online Resource 2. Risk of bias assessment of the SELECT and DECISION trials. [file 12885_2019_6369_MOESM2_ESM.docx]

# Online Resource 2. Risk of bias assessment of the SELECT and DECISION trials

| Parameter | SELECT  (Eisai Ltd 2017) | DECISION  (Bayer HealthCare 2017) |
| --- | --- | --- |
| Was the method used to assign participants to the treatment groups really random? | ✓ | ✓ |
| Was the allocation of treatment concealed? | ✓ | ✓ |
| Was the number of participants who were randomised stated? | ✓ | ✓ |
| Were details of baseline comparability presented in terms of prognostic factors? | ✓ | ✓ |
| Was baseline comparability achieved in terms of prognostic factors? | P ^a^ | P ^a^ |
| Were the eligibility criteria for study entry specified? | ✓ | ✓ |
| Were any co-interventions identified that may influence the outcomes for each group? | ✓ | ✓ |
| Were the outcome assessors blinded to the treatment allocation? | ✓ | ✓ |
| Were the individuals who administered the intervention blinded to the treatment allocation? | ✓ ^b^ | ✓ |
| Were the participants who received the intervention blinded to the treatment allocation? | ✓ ^c^ | ✓ ^d^ |
| Was the success of the blinding procedure assessed? |  |  |
| Were at least 80% of the participants originally included in the randomisation process followed up in the final analysis? | ✓ | ✓ |
| Were the reasons for withdrawals stated? | ✓ | ✓ |
| Is there any evidence to suggest that the authors measured more outcomes than they reported? | ✓ | ✓ |
| Was an intention to treat analysis included? | ✓ | ✓ |

✓ yes (item properly addressed) no (item not properly addressed) P partially (item partially addressed)

a In the SELECT trial, median time from diagnosis of DTC to randomisation was shorter in the lenvatinib arm than in the placebo arm (66.0 months versus 73.9 months). Compared with the placebo arm, a smaller proportion of patients in the lenvatinib arm had metastases in the lung (86.6% versus 94.7%) or liver (16.5% versus 21.4%). In the DECISION trial, a higher proportion of patients in the sorafenib arm had metastases in the lymph node (54.6%) or pleura (19.3%) than in the placebo arm (48.1% and 11.4% respectively).

b Study drugs administered by clinicians who remained unaware of the study-drug assignments until the occurrence of unacceptable toxic effects or disease progression as assessed by independent radiologic review

c If independent radiologic review confirmed disease progression, the patients who were receiving placebo could elect to enter the open-label lenvatinib phase

d In the event of protocol-defined progression determined by the investigator, treatment could be unmasked and patients from both groups could begin open-label sorafenib and continue until treatment was no longer beneficial, based on investigator judgment

# References

Bayer HealthCare (2017) Multiple technology appraisal. Lenvatinib and sorafenib for treating differentiated thyroid cancer after radioactive iodine. Company submission to NICE. March. <https://www.nice.org.uk/guidance/ta535/documents/committee-papers>. Accessed Aug 8 2018

Eisai Ltd (2017) Multiple technology appraisal [ID1059]. Lenvatinib for treating differentiated thyroid cancer after radioactive iodine. Eisai submission. April. <https://www.nice.org.uk/guidance/ta535/documents/committee-papers>. Accessed Aug 8 2018
