# Supplementary material for: A systematic review of lenvatinib and sorafenib for treating progressive, locally advanced or metastatic, differentiated thyroid cancer after treatment with radioactive iodine
Source: BMC Cancer. 2019 Dec 12;19:1209. doi: 10.1186/s12885-019-6369-7 (PMC6909631; doi:10.1186/s12885-019-6369-7)
Supplement: Supplementary file 3 — Additional file 3. Online Resource 3. Quality assessment of systematic review evidence included. [file 12885_2019_6369_MOESM3_ESM.docx]

# Online Resource 3. Quality assessment of systematic review evidence included

| Assessment criterion | Anderson et al. (2013) | Gruber & Colevas 2015 | Jean et al. (2016) | Kawalec et al. (2016) | McFarland & Misiukiewicz 2014 | Shen et al. (2014) | Thomas et al. (2014) | Trembaly et al. (2016) | Ye et al. (2015) | CADTH (2016) | CADTH (2015) | Eisai Ltd (2017) | Bayer HealthCare (2017) |
| --- | --- | --- | --- | --- | --- | --- | --- | --- | --- | --- | --- | --- | --- |
| Was the review question clearly defined in terms of population, interventions, comparators, outcomes and study designs? | ✓ | P | ✓ | ✓ | ✓ | ✓ | ✓ | ✓ | ✓ | ✓ | ✓ | ✓ | ✓ |
| Was the search strategy adequate and appropriate? | ✓ | ✓ | ^a^ | ✓ | ✓ | ✓ | ✓ | - | ✓ | ✓ | ✓ | ✓ | ✓ |
| Were preventative steps taken to minimise bias and errors in the study selection process? | ✓ | - | - | ✓ | ✓ | ✓ | - | - | ✓ | ✓ | ✓ | ✓ | ✓ |
| Were appropriate criteria used to assess the quality of the primary studies, and were preventative steps taken to minimise bias and errors in the quality assessment process | - | - | - | ^b^ | - |  |  | - | - | ✓^c^ | ✓^c^ | ✓ | ✓^d^ |
| Were preventative steps taken to minimise bias and errors in the data extraction process? | ✓ | - | - | ✓ | ✓ | ✓ | ✓ | - | ✓ | - | - | - | ✓ |
| Were adequate details presented for each of the primary studies? | ✓ | P | ✓ | ✓ | ✓ | ✓ | P | ✓ | ✓ | ✓ | ✓ | ✓ | ✓ |
| Were appropriate methods used for data synthesis? | ✓ | ✓ | ✓ | ✓ | ✓ | P^e^ | P^e^ | ✓ | P^f^ | ✓ | ✓ | ✓ | ✓ |
| Do the authors’ conclusions accurately reflect the evidence that was reviewed? | ✓ | ✓ | ✓ | ✓ | ✓ | ✓ | ✓ | ✓ | P^f^ | ✓ | ✓ | ✓ | ✓ |
| Was the review published in peer reviewed journal? | ✓ | ✓ | ✓ | ✓ | ✓ | ✓ | ✓ | ✓ | ✓ |  |  |  |  |
| Was the review sponsored by pharmaceutical company? | ✓^1^ |  |  |  |  |  | P^1^ | P^2^ |  |  |  | ✓^2^ | ✓^1^ |

✓ yes (item properly addressed) no (item not properly addressed) P partially (item partially addressed); - not reported

Key to sponsors: 1=Bayer HealthCare; 2=Eisai Ltd

a Only PubMed was searched

b Used the Jadad scale (not an appropriate assessment tool)

c Results of the assessment were not presented

d Only the DECISION trial was assessed

e No investigation of heterogeneity of studies included in meta-analysis

f Subgroup analyses were conducted based on patients with and without RR-DTC, however we consider that all studies of patients with DTC included a majority, if not all, of patients with RR-DTC

# References

Anderson RT, Linnehan JE, Tongbram V, Keating K, Wirth LJ (2013) Clinical, safety, and economic evidence in radioactive iodine-refractory differentiated thyroid cancer: a systematic literature review Thyroid 23:392-407 doi:<http://dx.doi.org/10.1089/thy.2012.0520>

Bayer HealthCare (2017) Multiple technology appraisal. Lenvatinib and sorafenib for treating differentiated thyroid cancer after radioactive iodine. Company submission to NICE. March. <https://www.nice.org.uk/guidance/ta535/documents/committee-papers>. Accessed Aug 8 2018

Canadian Agency for Drugs and Technologies in Health (CADTH) (2015) pan-Canadian Oncology Drug Review Final Clinical Guidance Report Sorafenib (Nexavar) for Differentiated Thyroid Cancer. CADTH. <https://www.cadth.ca/sites/default/files/pcodr/pcodr_sorafenib_nexavar_dtc_fn_cgr.pdf>. Accessed May 16 2017

Canadian Agency for Drugs and Technologies in Health (CADTH) (2016) pan-Canadian Oncology Drug Review Final Clinical Guidance Report: Lenvatinib (Lenvima) for Differentiated Thyroid Cancer. CADTH. <https://www.cadth.ca/sites/default/files/pcodr/pcodr_lenvatinib_lenvima_dtc_fn_cgr.pdf>. Accessed May 16 2017

Eisai Ltd (2017) Multiple technology appraisal [ID1059]. Lenvatinib for treating differentiated thyroid cancer after radioactive iodine. Eisai submission. April. <https://www.nice.org.uk/guidance/ta535/documents/committee-papers>. Accessed Aug 8 2018

Gruber JJ, Colevas AD (2015) Differentiated thyroid cancer: focus on emerging treatments for radioactive iodine-refractory patients Oncologist 20:113-126 doi:<http://dx.doi.org/10.1634/theoncologist.2014-0313>

Jean GW, Mani RM, Jaffry A, Khan SA (2016) Toxic Effects of Sorafenib in Patients With Differentiated Thyroid Carcinoma Compared With Other Cancers JAMA Oncol 2:529-534 doi:<http://dx.doi.org/10.1001/jamaoncol.2015.5927>

Kawalec P, Malinowska-Lipien I, Brzostek T, Kozka M (2016) Lenvatinib for the treatment of radioiodine-refractory differentiated thyroid carcinoma: a systematic review and indirect comparison with sorafenib Expert Rev Anticancer Ther 16:1303-1309 doi:10.1080/14737140.2016.1247697

McFarland DC, Misiukiewicz KJ (2014) Sorafenib in radioactive iodine-refractory well-differentiated metastatic thyroid cancer Onco Targets Ther 7:1291-1299 doi:<http://dx.doi.org/10.2147/OTT.S49430>

Shen CT, Qiu ZL, Luo QY (2014) Sorafenib in the treatment of radioiodine-refractory differentiated thyroid cancer: a meta-analysis Endocr Relat Cancer 21:253-261 doi:<http://dx.doi.org/10.1530/ERC-13-0438>

Thomas L, Lai SY, Dong W, Feng L, Dadu R, Regone RM, Cabanillas ME (2014) Sorafenib in metastatic thyroid cancer: a systematic review Oncologist 19:251-258 doi:<http://dx.doi.org/10.1634/theoncologist.2013-0362>

Tremblay G, Holbrook T, Milligan G, Pelletier C, Rietscheli P (2016) Matching-adjusted indirect treatment comparison in patients with radioiodine-refractory differentiated thyroid cancer Comp Eff Res 6:13-21

Ye X, Zhu Y, Cai J (2015) Relationship between toxicities and clinical benefits of newly approved tyrosine kinase inhibitors in thyroid cancer: A meta-analysis of literature J Cancer Res Ther 11 Suppl 2:C185-190 doi:<http://dx.doi.org/10.4103/0973-1482.168182>
